# Supplementary material for: Inflammation promotes resistance to immune checkpoint inhibitors in high microsatellite instability colorectal cancer
Source: Nat Commun. 2022 Nov 28;13:7316. doi: 10.1038/s41467-022-35096-6 (PMC9705377; doi:10.1038/s41467-022-35096-6)
Supplement: Supplementary file 1 — Supplementary Information [file 41467_2022_35096_MOESM1_ESM.pdf]

Inflammations promote resistance to immune checkpoint inhibitors in high microsatellite instability colorectal cancer

Supplementary Figures

Supplementary Figure 1

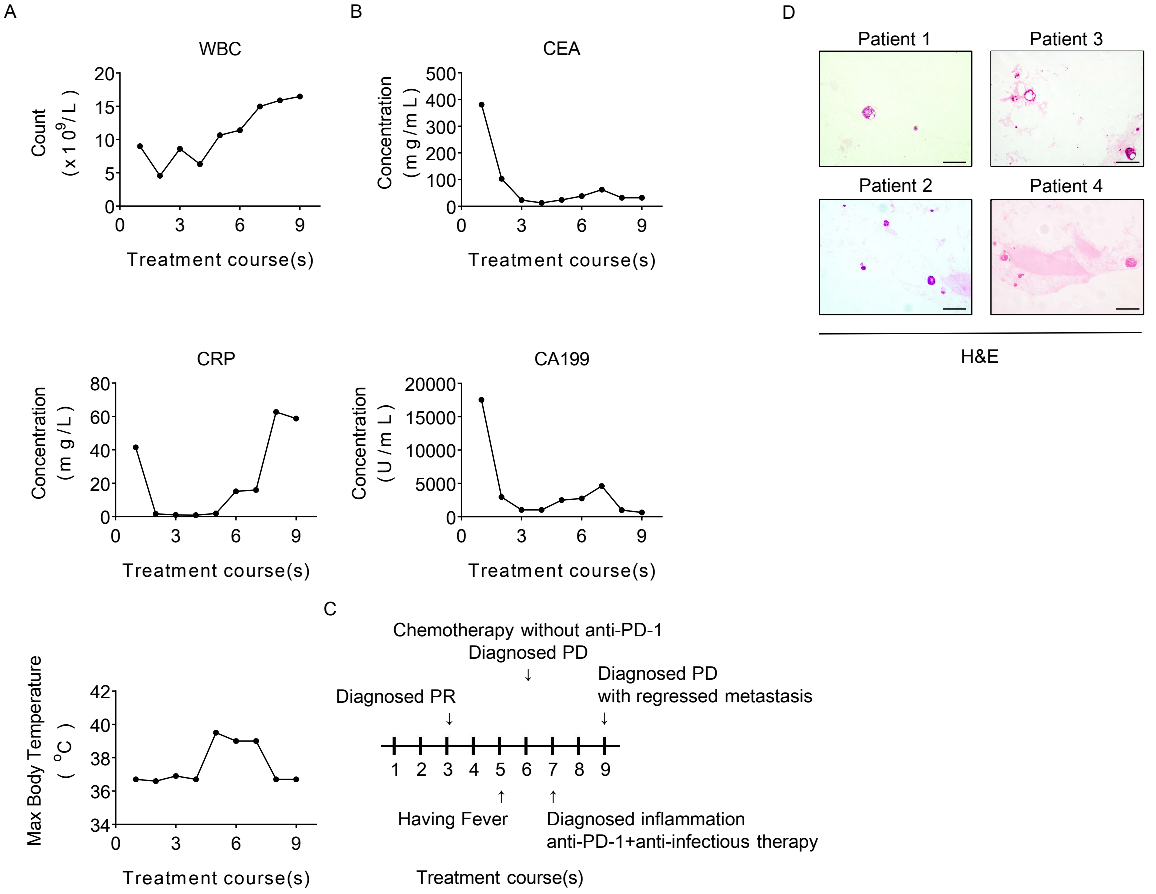

Supplementary Figure 1. Clinical data of Patient 1 and images of organoid. (A) White blood cell (WBC) count, C-reactive protein (CRP) concentration and max body temperature of Patient 1 prior to treatment in each course of Pembrolizumab. (B) Blood carcino-embryonic antigen (CEA) and carbohydrate antigen 199 (CA199) concentration of Patient 1 prior to treatment in each course of Pembrolizumab. (C) Summary of the diagnostic and therapeutic process of Patient 1. (D) H&E staining images (Scale bar: 100 μm) of tumor organoids from 4 patients are shown. Each experiment was repeated 3 times.

Supplementary Figure 2

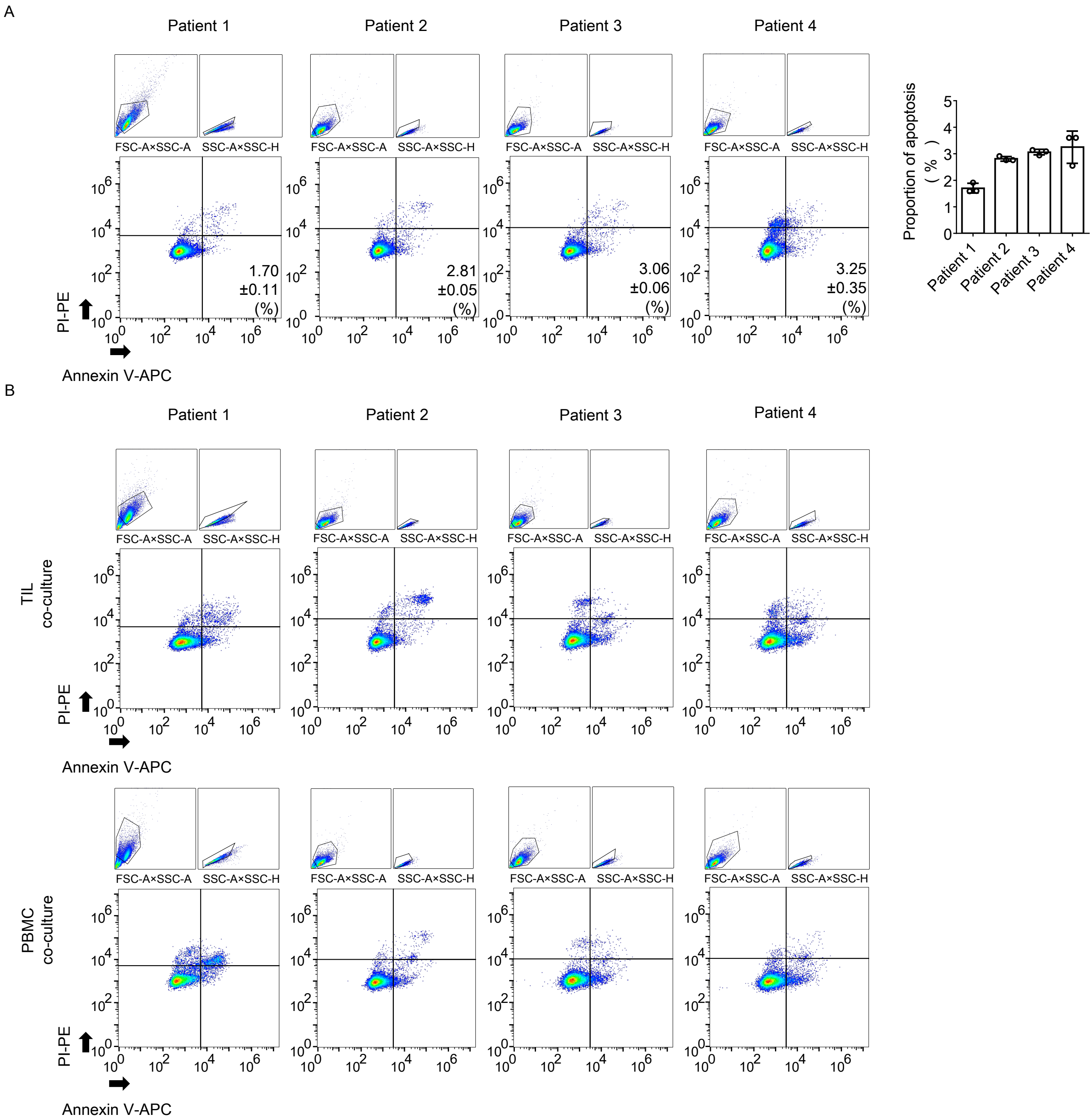

Supplementary Figure 2. Gating strategies of apoptosis assays of organoids.

(A) Tumor organoids prior to co-culture were separated for apoptosis assays. The mean values are shown, and the standard deviations are displayed by the error bars. Gating strategies are shown. Each experiment was repeated 3 times with 3 replicates. (B) Gating strategies of apoptosis assays of Figure 1E are shown.

Supplementary Figure 3

A

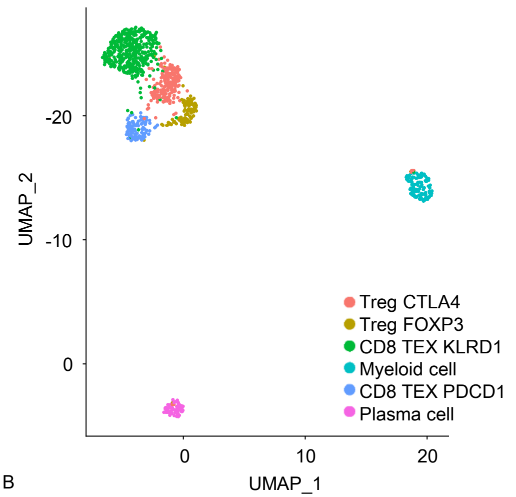

Cell type	Marker
Treg CTLA4	PTPRC, CD3D/E/G, CD4, CTLA4
Treg FOXP3	PTPRC, CD3D/E/G, CD4, FOXP3, CTLA4
CD8 TEX KLRD1	PTPRC, CD3D/E/G, CD8A, CD69, CTLA4, HAVER2, GZMB, IFNG, KLRD1
CD8 TEX PDCD1	PTPRC, CD3D/E/G, CD8A/B, CD69, CTLA4, HAVER2, GZMB, IFNG,PDCD1
Myeloid cell	PTPRC, C1QB, AIF, LYZ
Plasma cell	PTPRC, MZB1, IGLL5

B

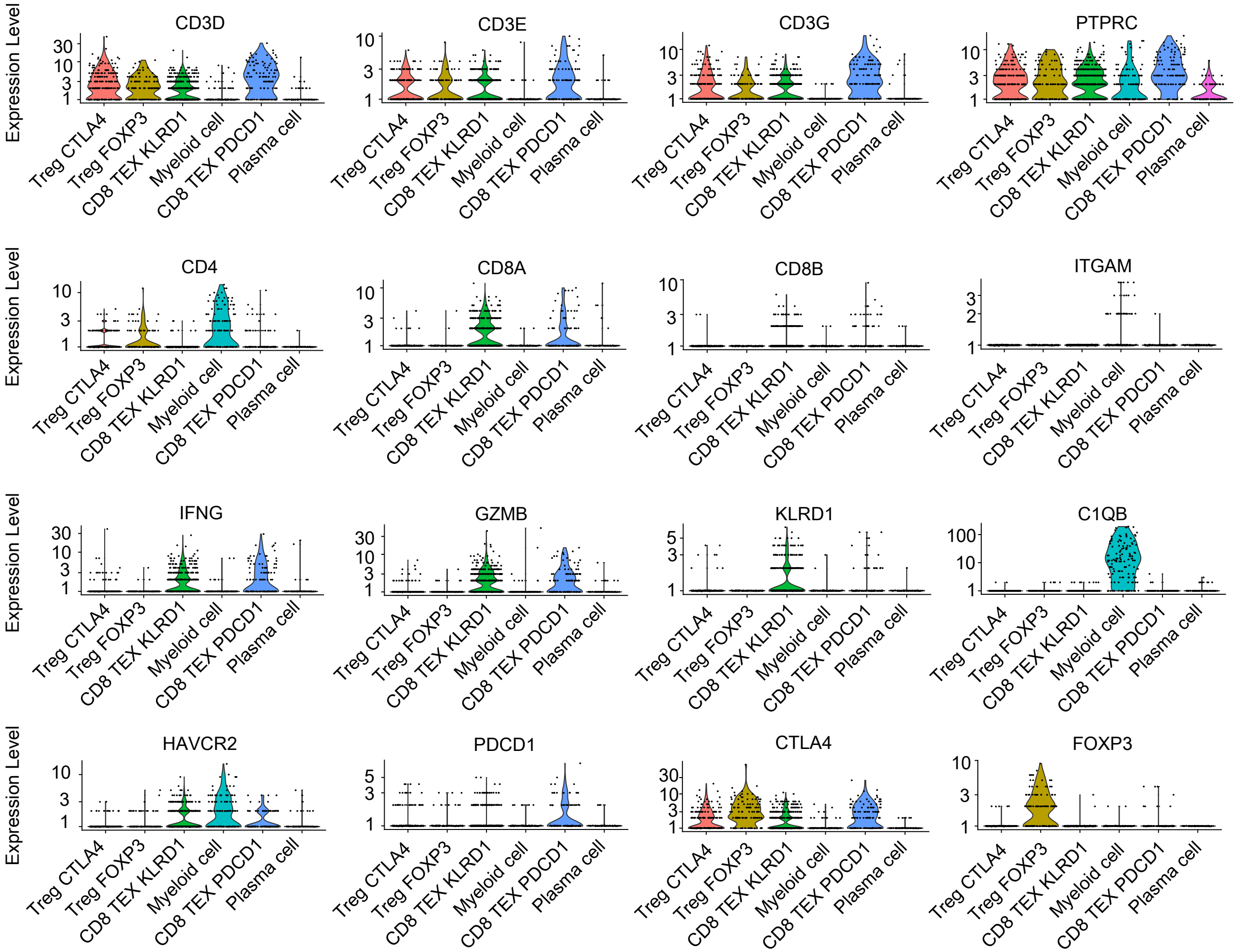

Supplementary Figure 3. Clustering information of immune cells in single-cell sequencing of Patient 1. (A)The immune cells were extracted and clustered using Seurat (v3) software. The cells were divided into six subtypes, including two Treg cell clusters (Treg CTLA4 and Treg FOXP3), two $CD8^{+}$ cell clusters (CD8 TEX KLRD1 and CD8 TEX PDCD1), a plasma cell cluster and a myeloid cell cluster. (B) The expression level of immune cell marker genes in each cluster is shown using a violin plot. $CD8^{+}$ T cells were mainly exhausted cells expressing *PDCD1* or *KLRD*, and $CD4^{+}$ T cells were mainly Treg cells characterized by *FOXP3* expression with or without *CTLA4* expression. Myeloid cells were identified by high expression of *PTPRC, C1QB, AIF*, and *LYZ*. Plasma cells were identified by high expression of *PTPRC, MZB1* and *IGLL5*.

Supplementary Figure 4

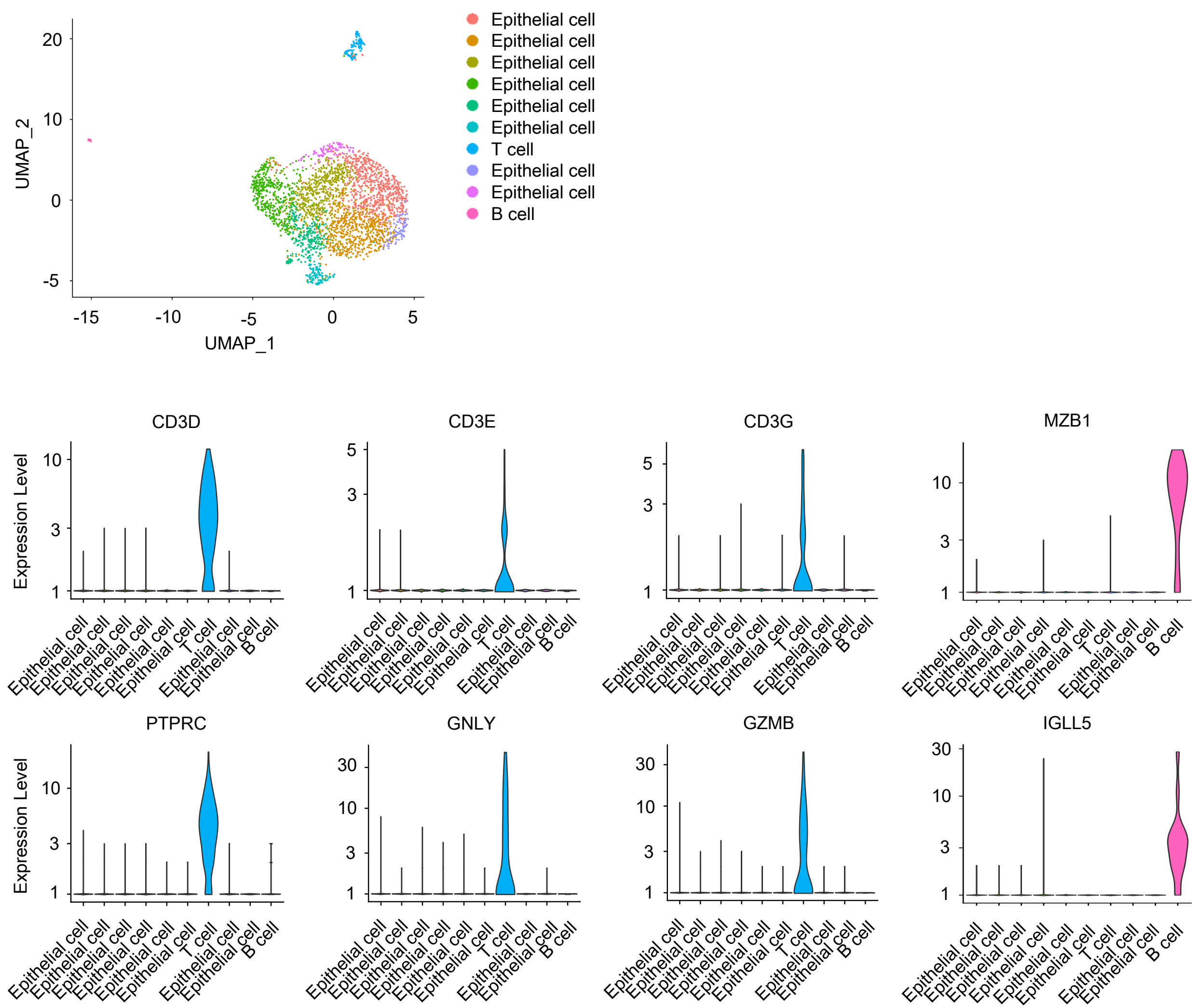

Supplementary Figure 4. Clustering information of single-cell sequencing of Patient 2. The 3088 qualified cells from Patient 2 were divided into 5 epithelial cells and 2 groups of immune cells using a UMAP plot. The expression level of marker genes in each cluster is shown using a violin plot.

Supplementary Figure 5

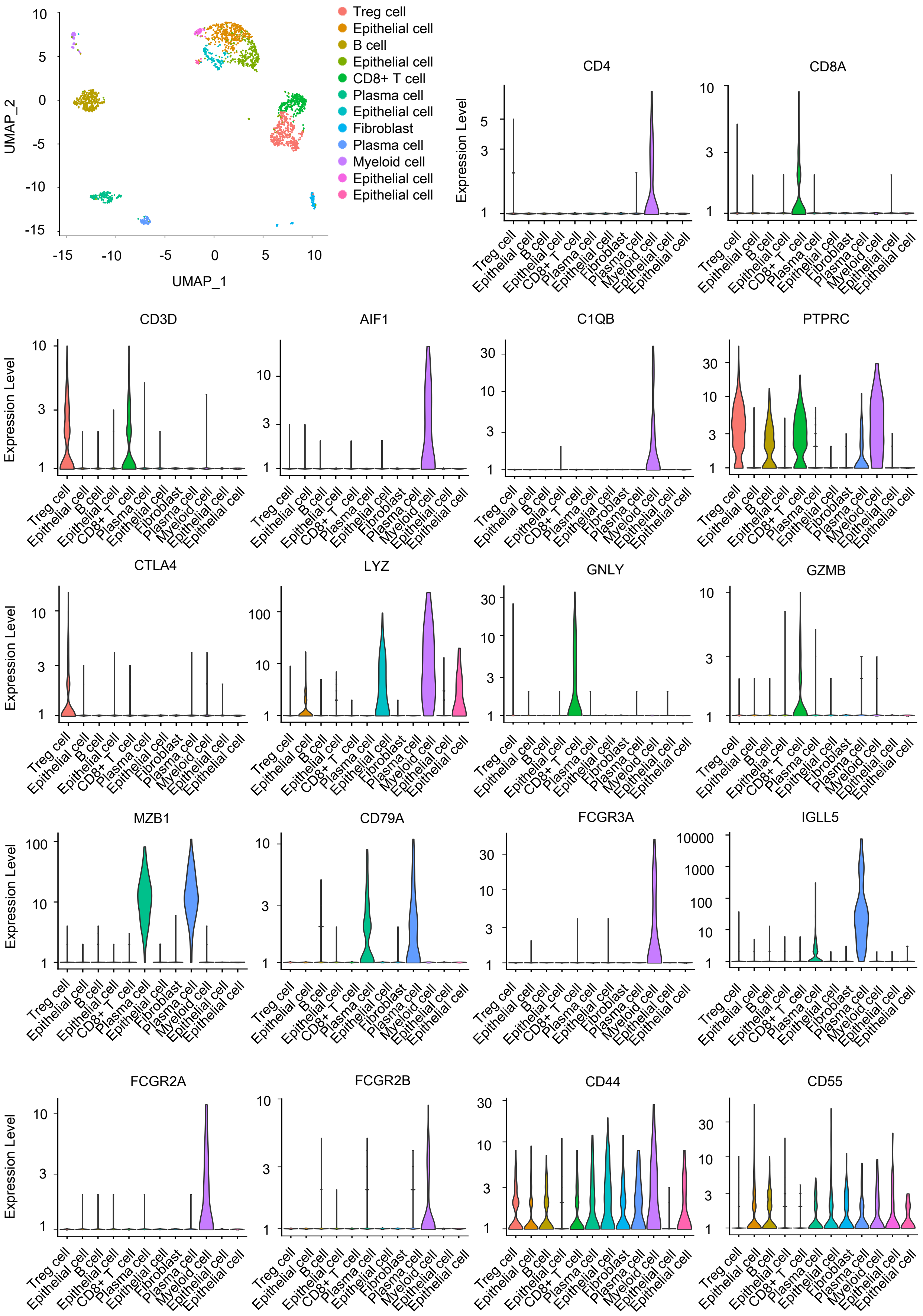

Supplementary Figure 5. Clustering information of single-cell sequencing of Patient 3. The 2166 qualified cells from Patient 3 were divided into 5 epithelial cells, Treg cell, B cell, plasma cell, $CD8^+$ T cell, fibroblast and myeloid cell using a UMAP plot. The expression level of marker genes in each cluster is shown using a violin plot.

Supplementary Figure 6

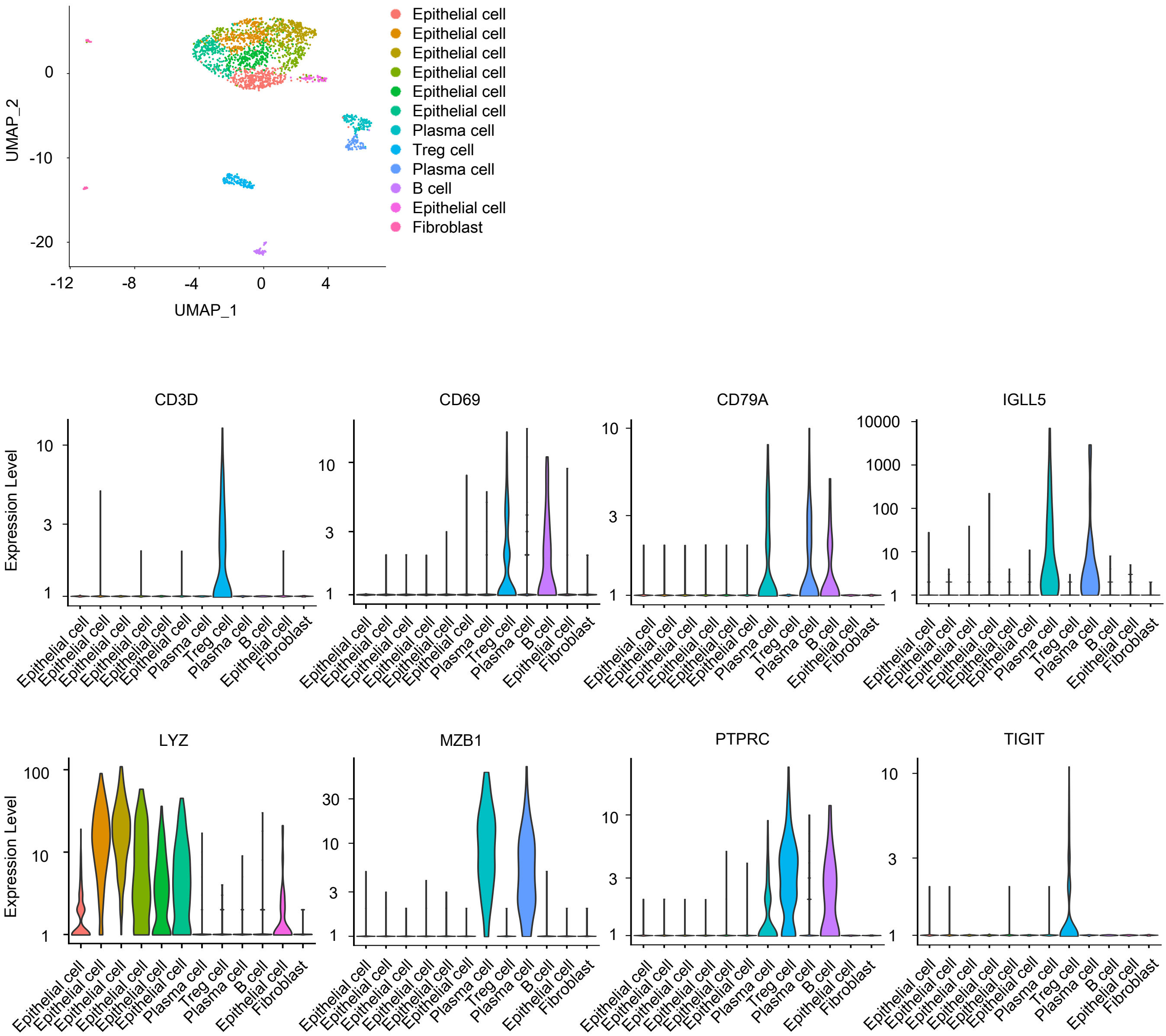

Supplementary Figure 6. Clustering information of single-cell sequencing of Patient 4. The 2375 qualified cells from Patient 4 were divided into 7 epithelial cells, plasma cell, B cell, Treg cell and fibroblast using a UMAP plot. The expression level of marker genes in each cluster is shown using a violin plot.

Supplementary Figure 7

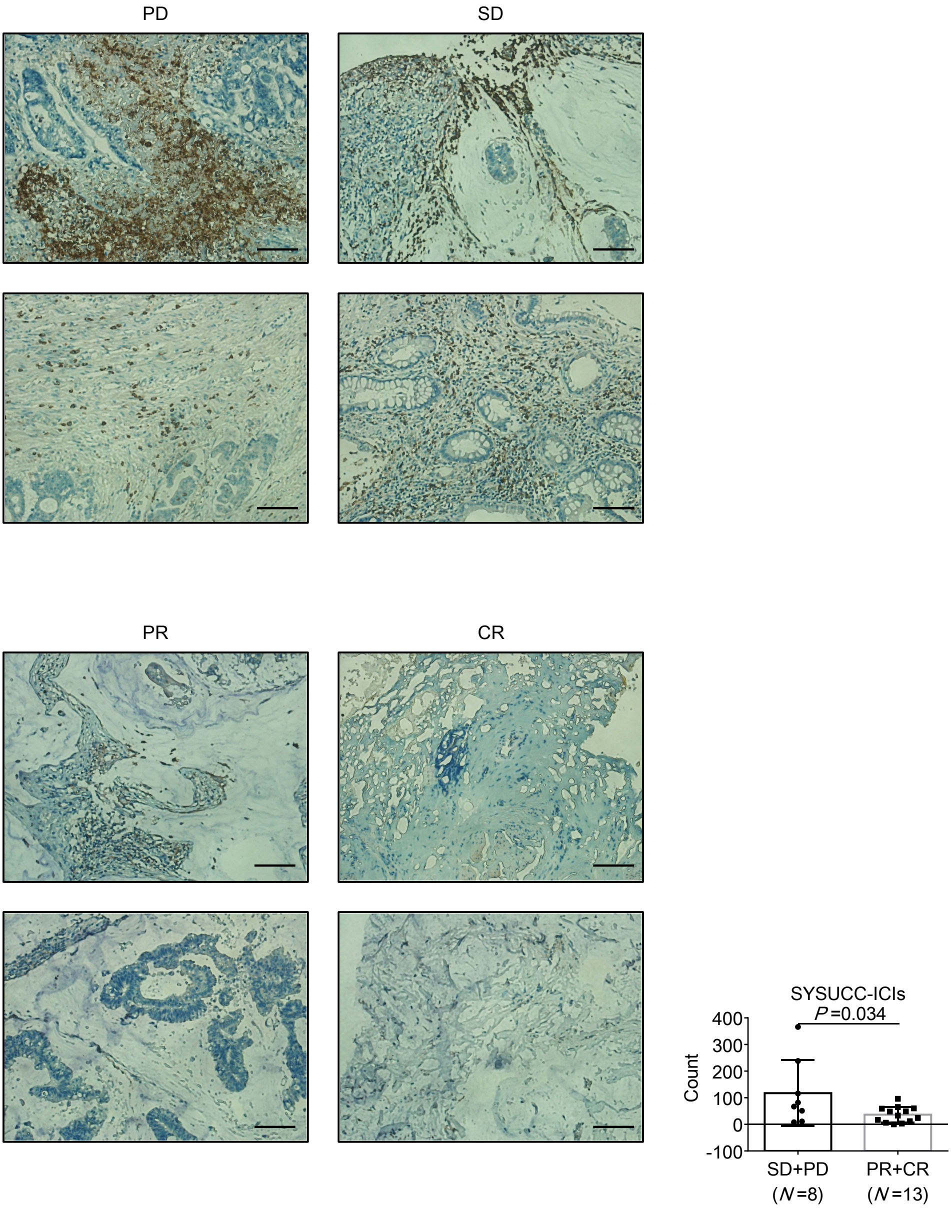

Supplementary Figure 7. IHC examinations for CD11b. Typical images of IHC examinations for CD11b among patients diagnosed CR, PR, SD and PD are shown (scale bars: 100μm). the average counting for $CD11b^{+}$ cell were compared between patients diagnosed SD or PD and those diagnosed CR or PR. Two-tailed unpaired Student's *t* test was used and the mean value and the standard deviation is displayed. The average number of positive cells in 5 selected fields in the tumor area or previous tumor site was counted for each patients. PR: partial response; CR: complete response; SD: stable disease; PD: progressed disease.

Supplementary Figure 8

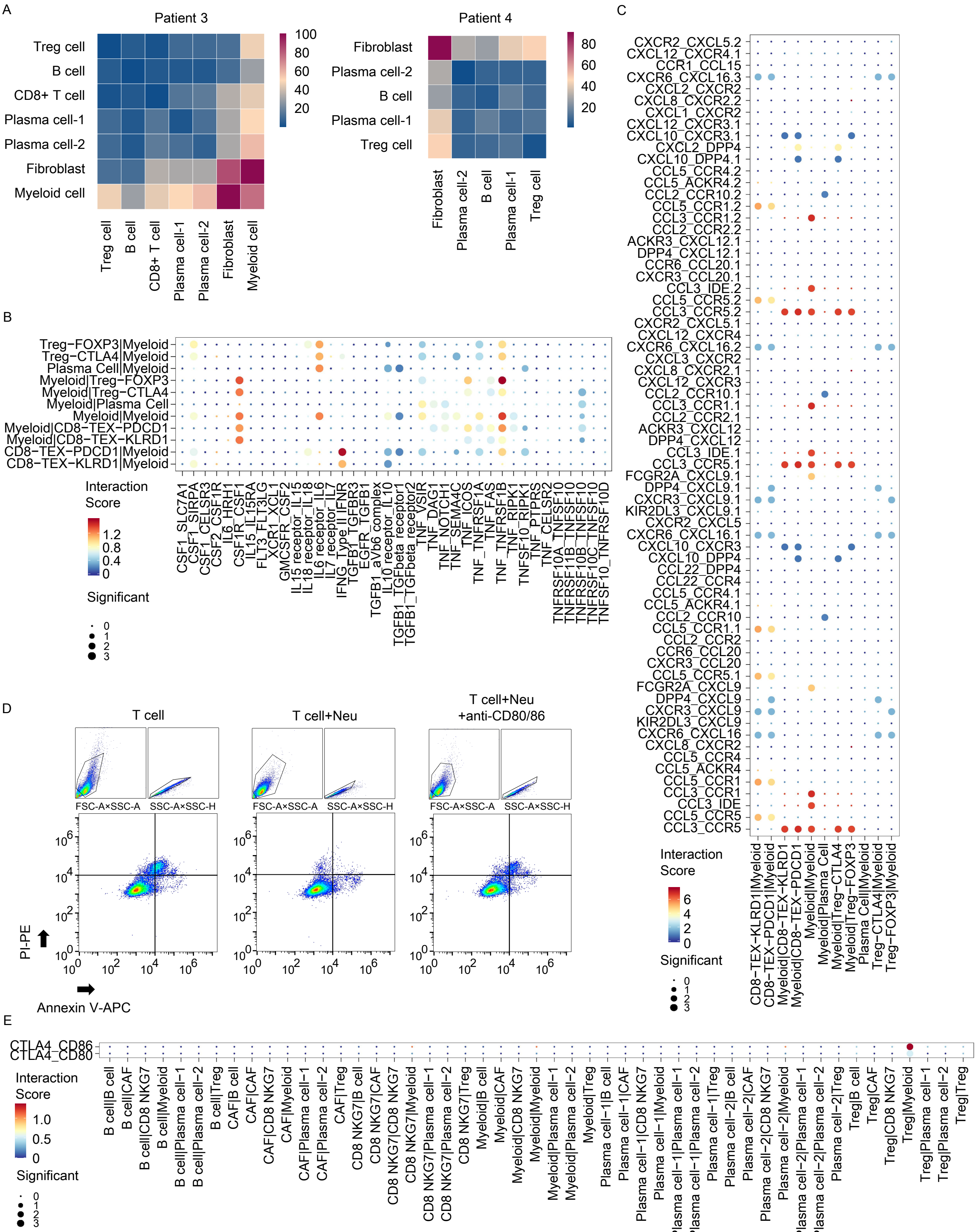

Supplementary Figure 8. Analysis of interactions and ligand-receptor interactions, and gating strategies of apoptosis assays of Figure 3E. (A) Interactions among subtypes of immune cells and fibroblast were analyzed in Patient 3 and 4. Functional phenotypes and predicted interactions between cells are shown. (B-C) Predicted ligand-receptor interaction of chemokines and cytokines between subtypes of immune cells in Patient 1 are shown. (D) Gating strategies of apoptosis assays of Figure 3E are shown. (E) Ligand-receptor interactions in CD80/CD86-CTLA4 axis between immune cells in Patient 3 are shown.

Supplementary Figure 9

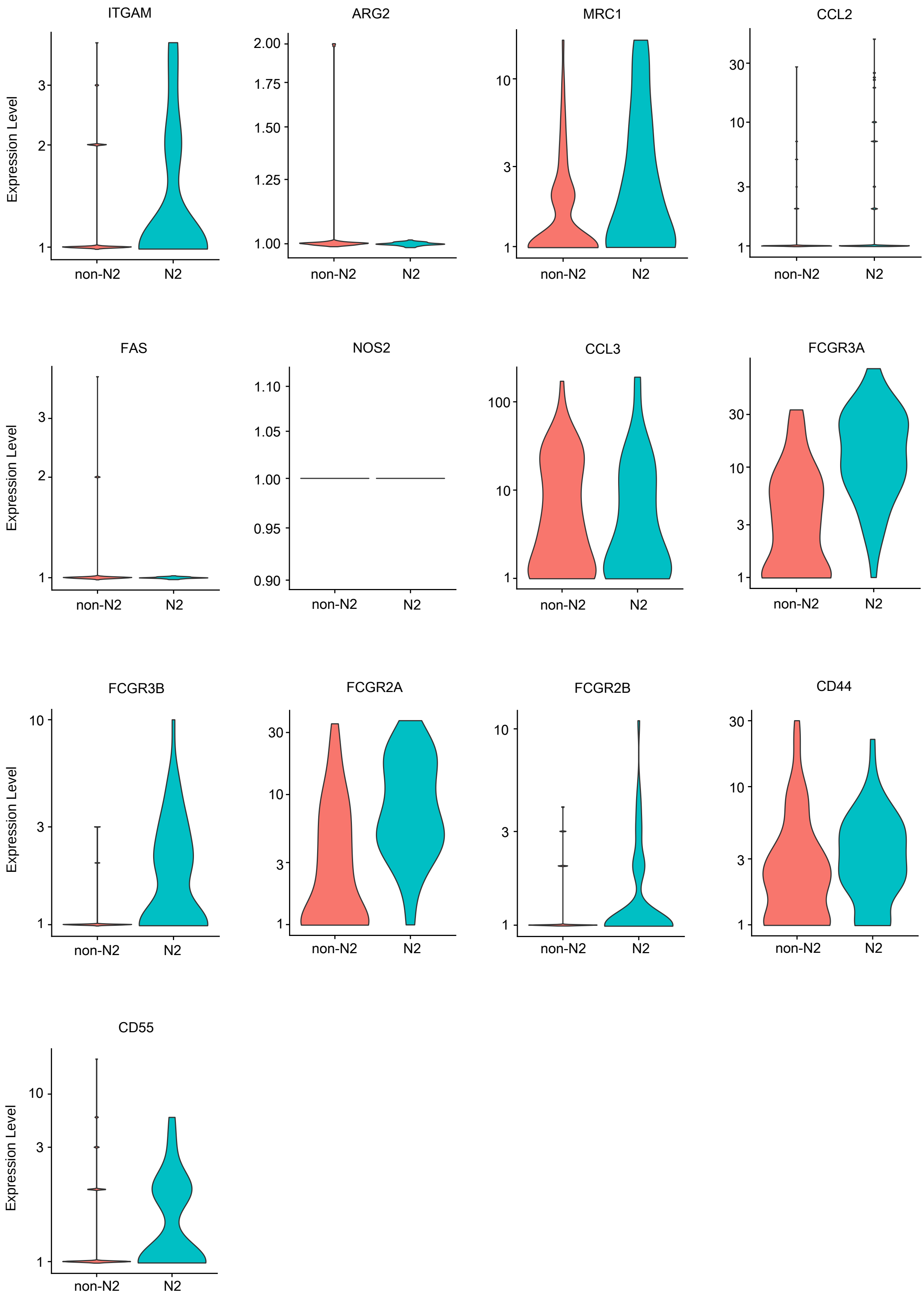

Supplementary Figure 9. Expression level of N1 and N2-associated marker genes in myeloid cells of Patient 1. The expression level of marker genes of N1 and N2 clusters, and function markers of neutrophils in each cluster are shown using a violin plot.

Supplementary Figure 10

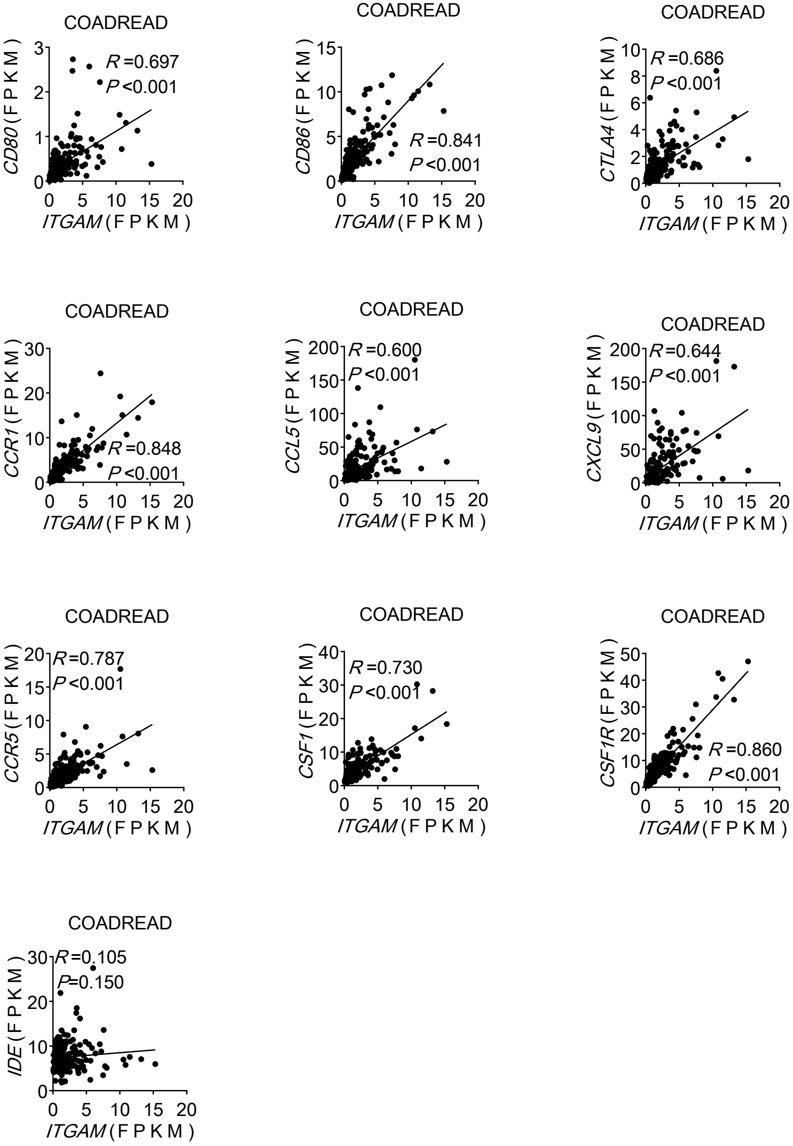

Supplementary Figure 10. Correlations between expression level of *ITGAM* and immune-associated markers in MSI CRCs in TCGA. The correlations between expression level of *ITGAM* and the other immune-associated ligands and receptors in TCGA are shown. The Spearman rank correlation test was used.
