# Supplementary material for: Inflammation promotes resistance to immune checkpoint inhibitors in high microsatellite instability colorectal cancer
Source: Nat Commun. 2022 Nov 28;13:7316. doi: 10.1038/s41467-022-35096-6 (PMC9705377; doi:10.1038/s41467-022-35096-6)
Supplement: Supplementary file 3 — Reporting Summary [file 41467_2022_35096_MOESM3_ESM.pdf]

nature portfolio

Corresponding author(s): DBPR-NCOMMS-21-35525B

Last updated by author(s): Nov 3, 2022

Reporting Summary

Nature Portfolio wishes to improve the reproducibility of the work that we publish. This form provides structure for consistency and transparency in reporting. For further information on Nature Portfolio policies, see our Editorial Policies and the Editorial Policy Checklist.

Statistics

For all statistical analyses, confirm that the following items are present in the figure legend, table legend, main text, or Methods section.

n/a	Confirmed	
☐	☒	The exact sample size (*n*) for each experimental group/condition, given as a discrete number and unit of measurement
☐	☒	A statement on whether measurements were taken from distinct samples or whether the same sample was measured repeatedly
☐	☒	The statistical test(s) used AND whether they are one- or two-sided<br>*Only common tests should be described solely by name; describe more complex techniques in the Methods section.*
☐	☒	A description of all covariates tested
☐	☒	A description of any assumptions or corrections, such as tests of normality and adjustment for multiple comparisons
☐	☒	A full description of the statistical parameters including central tendency (e.g. means) or other basic estimates (e.g. regression coefficient) AND variation (e.g. standard deviation) or associated estimates of uncertainty (e.g. confidence intervals)
☐	☒	For null hypothesis testing, the test statistic (e.g. *F*, *t*, *r*) with confidence intervals, effect sizes, degrees of freedom and *P* value noted<br>*Give P values as exact values whenever suitable.*
☒	☐	For Bayesian analysis, information on the choice of priors and Markov chain Monte Carlo settings
☐	☒	For hierarchical and complex designs, identification of the appropriate level for tests and full reporting of outcomes
☐	☒	Estimates of effect sizes (e.g. Cohen's *d*, Pearson's *r*), indicating how they were calculated

Our web collection on statistics for biologists contains articles on many of the points above.

Software and code

Policy information about availability of computer code

Data collection	An scRNA-seq library was prepared using the DNBelab C4 system. DIPSEQ T1 was used for sequencing libraries at the National Gene Bank (CNGB, BGI-SHENZHEN, Shenzhen, China). The raw FASTQ files were processed by DNBelab_C_Series_HT_scRNA-analysis-software (https://github.com/MGI-tech-bioinformatics/DNBelab_C_Series_HT_scRNA-analysis-software). Briefly, the FASTQ raw data were converted to a Cell Ranger-specific FASTQ file, then the converted FASTQ files were aligned to GRCH38 human reference using STAR software (v2.5.3). Cell clustering was conducted by the Seurat (v3.1)52 package in RStudio (v1.1.383). Cell apoptosis analysis was conducted using a Beckman CytoFLEX FCM (Beckman Coulter) with CytExpert 2.0. The proportion of apoptosis was calculated using FlowJo V10 (BD).
Data analysis	The FASTQ files were processed separately using a modified version of the Cell Ranger count pipeline, which aligned cDNA reads with the GRCH38 human reference using STAR software (v2.5.3).Cell clustering was conducted by the Seurat (v3.1) package in RStudio. To analyze cell-to-cell interactions, we used CellPhoneDB46 to identify significant ligand-receptor pairs in samples. SPSS 19.0 (Chicago, IL) and GraphPad Prism 6 (San Diego, CA) were used for statistical analysis.

For manuscripts utilizing custom algorithms or software that are central to the research but not yet described in published literature, software must be made available to editors and reviewers. We strongly encourage code deposition in a community repository (e.g. GitHub). See the Nature Portfolio guidelines for submitting code & software for further information.

Data

Policy information about availability of data

All manuscripts must include a data availability statement. This statement should provide the following information, where applicable:

- Accession codes, unique identifiers, or web links for publicly available datasets
- A description of any restrictions on data availability
- For clinical datasets or third party data, please ensure that the statement adheres to our policy

The raw scRNA-seq data in this study have been deposited at the Gene Expression Omnibus (GEO) database under accession code GSE179784 [https://www.ncbi.nlm.nih.gov/geo/query/acc.cgi?acc=GSE179784]. The GRCH38 human reference are available at UCSC Genome Browser Home [http://genome.ucsc.edu/]. The referenced raw data from TCGA are available at TCGA database [https://portal.gdc.cancer.gov/], and processed data are available within the Source data. The clinical data and experiment data in this study are provided in the Source Data file. A reporting summary for this article is available as a Supplementary Information file. Source data are provided with this paper.

Field-specific reporting

Please select the one below that is the best fit for your research. If you are not sure, read the appropriate sections before making your selection.

☒ Life sciences ☐ Behavioural & social sciences ☐ Ecological, evolutionary & environmental sciences

For a reference copy of the document with all sections, see nature.com/documents/nr-reporting-summary-flat.pdf

Life sciences study design

All studies must disclose on these points even when the disclosure is negative.

Sample size	No sample size calculation was performed. The retrospective study included all of the patients who met the inclusion criteria in our center. Patients diagnosed MSI-H CRC from Sun Yat-sen University Cancer Center (SYSUCC, Guangzhou, China) who started to receive PD-1 blockade therapy from January 2017 to October 2020 were retrospectively enrolled. The inclusion criteria were as follows: 1) pathologically diagnosed colorectal cancer; 2) genetically diagnosed MSI-H; 3) receiving PD-1 blockade. The exclusion criteria were as follows: 1) receiving only postoperative PD-1 blockade after radical surgery; 2) receiving less than 2 courses of treatment; 3) without determination of tumor response. Sixty eight patients were included, while 4 patients were excluded for receiving only postoperative treatment, 1 for receiving 1 course of treatment and 1 for without determination of tumor response in our center. Finally, 62 patients were enrolled. In addition, 142 previously reported dMMR/MSI-H CRC patients who received surgical treatment with preoperative blood test data and sufficient tumors for counting tumor-infiltrating lymphocytes (TILs) were retrospectively enrolled (Liu, et al. J Natl Cancer I. 2018).
Data exclusions	Among patients with MSI-H CRC who received PD-1 blockade, the exclusion criteria were as follows: 1) receiving only postoperative PD-1 blockade after radical surgery; 2) receiving less than 2 courses of treatment; 3) without determination of tumor response. Sixty eight patients were included, while 4 patients were excluded for receiving only postoperative treatment, 1 for receiving 1 course of treatment and 1 for without determination of tumor response in our center. Finally, 62 patients were enrolled. The exclusion criteria were pre-established.<br>For NLR calculation, 4 patients without consistent blood test data were excluded. The exclusion was not pre-established.
Replication	Each apoptosis assays contained 3 replicates and was repeated 3 times. The sample of each replicates was collected from individual wells, and the experiments were conducted 3 times independently. For organoids, the apoptotic proportions were comparable between TIL and PBMC groups among samples from Patient 2-4, and were higher in PBMC group in samples from Patient 1. For MC38, the apoptotic proportions were decreased in T cell+neu group compared to T cell group, while anti-CD80/CD86 treatment could rescue the apoptotic proportions.<br>For lymphocyte and neutrophil counting, the number of lymphocytes in high-power fields (HPF) was counted by a pathologist according to the following method: select five HPFs; count the positive cells; and take the average.
Randomization	Murine T cells were divided into three parts and randomly allocated into PD-1 blockade; PD-1 blockade plus neutrophil co-culture, and PD-1 blockade plus CD80/CD86-neutralized neutrophil co-culture. The randomization is not relevant to the other experiments in the current study. Specified organoid cells and paired T cells were used in the organoid-T cell co-culture experiments.
Blinding	For experiments, the name of each groups were marked with paired numbers, and researchers who conducted data collection and analysis were blinded to groups. Meanwhile, researchers who conduct experiments did not participate in data collection and analysis. For retrospective clinical investigations, researchers who conduct grouping did not participate in data analysis, and researchers were blinded to groups were analyzing clinical data.

Reporting for specific materials, systems and methods

We require information from authors about some types of materials, experimental systems and methods used in many studies. Here, indicate whether each material, system or method listed is relevant to your study. If you are not sure if a list item applies to your research, read the appropriate section before selecting a response.

Materials & experimental systems

n/a	Involved in the study	
☐	☒	Antibodies
☐	☒	Eukaryotic cell lines
☒	☐	Palaeontology and archaeology
☐	☒	Animals and other organisms
☐	☒	Human research participants
☐	☒	Clinical data
☒	☐	Dual use research of concern

Methods

n/a	Involved in the study	
☒	☐	ChIP-seq
☐	☒	Flow cytometry
☒	☐	MRI-based neuroimaging

Antibodies

Antibodies used

Anti-human CD3 antibody (Peprotech, clone OKT-3, 05121-25-500, 5 μg/mL dilution) and anti-human CD28 antibody (Peprotech, clone CD28.2, 10311-25-500, 5 μg/mL dilution) for stimulating human T cells; Anti-mouse CD3 antibody (Peprotech, clone 17A2, 05112-25-500, 1 μg/mL dilution), anti-mouse CD28 antibody (Peprotech, clone 37.51, 10312-25-1000, 1 μg/mL dilution), anti-mouse PD-1 neutralizing antibody (BioXcell, clone 29F.1A12, BE0273, 1 μg/mL dilution) , anti-mouse CD80 neutralizing antibody (R&D system, AF740-SP, 2 μg/mL dilution) and anti-mouse CD86 neutralizing antibody (R&D system, MAB741-SP, 2 μg/mL dilution) were used for stimulating murine neutrophils. Anti-human CD11b IgG monoclonal antibody (Abcam, clone EP1344, ab133357, 1/4000 dilution) was used for IHC staining. The other primary and secondary antibodies used for IHC examination and TIL counting were reported in our previous study (Liu, et al. J Natl Cancer I. 2018): Anti-human CD4 IgG monoclonal antibody (ORIGENE, clone 1F6, product not available, 1/50 dilution), Anti-human CD8 IgG monoclonal antibody (ORIGENE, clone SP16, DRM012, 1/80 dilution) and Anti-human FOXP3 IgG monoclonal antibody(Abcam, clone 236A/E7, ab20034, 1/100 dilution), anti-rabbit/mouse IgG monoclonal antibody (DAKO Real Envision).

Validation

CD11b: Validated for IHC.
Abcam website statement:
Positive control: IHC-P: Human tonsil and spleen tissues; Rat cerebrum and bone marrow tissue; Mouse lung and colon tissue. The Abpromise guarantee: IHC-P: 1/4000. Perform heat mediated antigen retrieval with citrate buffer pH 6 before commencing with IHC staining protocol. Please optimize IHC protocol when testing mouse and rat tissues. It is easy to show background staining in liver tissue. Reference: Michalon A, et al. Nat Commun 12:3142 (2021).
The other primary and secondary antibodies used for TIL counting in our previous studies has been reported (Liu, et al. J Natl Cancer I. 2018).

Eukaryotic cell lines

Policy information about cell lines

Cell line source(s)

MC38 cells from colon cancer of C57BL/6 mouse.

Authentication

None of the cell line used were authenticated.

Mycoplasma contamination

All the cell lines tested negative for the Mycoplasma contamination.

Commonly misidentified lines
(See ICLAC register)

No commonly misidentified cell lines were used in the study.

Animals and other organisms

Policy information about studies involving animals; ARRIVE guidelines recommended for reporting animal research

Laboratory animals

The study did not involve laboratory animals. T cells and neutrophils from C57BL/6 mice were previously preserved by liquid nitrogen.

Wild animals

The study did not involve wild animals.

Field-collected samples

The study did not involve field-collected samples.

Ethics oversight

No ethical approval was required since the study did not involve animals.

Note that full information on the approval of the study protocol must also be provided in the manuscript.

Human research participants

Policy information about studies involving human research participants

Population characteristics

Patients with MSI-H CRC from Sun Yat-sen University Cancer Center (SYSUCC, Guangzhou, China) who received ICI treatment were enrolled. There were 42 males and 20 females included. The age was ranged from 19 to 73. All those patients received at least 2 courses of ICIs and had evaluations of tumor response prior to surgery. Another 142 MSI-H CRCs who received

	surgical resection were enrolled as previously described (Liu, et al. J Natl Cancer I. 2018).
Recruitment	We retrospectively enrolled the MSI-H CRC patients who started to receive PD-1 blockade therapy from January 2017 to October 2020 in our center. The inclusion criteria were as follows: 1) pathologically diagnosed colorectal cancer; 2) genetically diagnosed MSI-H; 3) receiving PD-1 blockade. The exclusion criteria were as follows: 1) receiving only postoperative PD-1 blockade after radical surgery; 2) receiving less than 2 courses of treatment; 3) without determination of tumor response. Four patients were excluded for receiving only postoperative treatment, 1 for receiving 1 course of treatment and 1 for without determination of tumor response in our center. Finally, 62 patients were enrolled. The sample size is limited, which could lead to admission rate bias and cause type I error.
Ethics oversight	All procedures performed in studies involving human participants were approved by the ethical standards of the Ethics Committee of Sun Yat-sen University Cancer Center (GZR2020-273) and were in accordance with the 1964 Helsinki declaration and its later amendments or comparable ethical standards.

Note that full information on the approval of the study protocol must also be provided in the manuscript.

Clinical data

Policy information about clinical studies

All manuscripts should comply with the ICMJE guidelines for publication of clinical research and a completed CONSORT checklist must be included with all submissions.

Clinical trial registration	The current research does not contain clinical trial.
Study protocol	The current research does not contain clinical trial.
Data collection	Data was retrospectively collected using the tracking system in the center. Patients who met the inclusion criteria from January 2017 to Octorber 2020 were enrolled. The last follow-up date was June 14, 2022.
Outcomes	Tumor responses were determined as complete response (CR), partial response (PR), stable disease (SD) and progressive disease (PD) by radiologists according to the tracking system. Pathological tumor responses were determined using TRG grading scale by pathologists. TRG 0 is defined as no residual tumor cells found microscopically on multiple consecutive sections. TRG 1 is defined as the presence of only small clusters of tumor cells that can be observed under the plasma membrane. TRG 2 is defined as fibrosis within the tumor lesion and the observation of fragmented residual tumor cells. TRG 3 was defined as a lesion with little to no fibrosis and no change in the number of tumor cells.

Flow Cytometry

Plots

Confirm that:

☒ The axis labels state the marker and fluorochrome used (e.g. CD4-FITC).

☒ The axis scales are clearly visible. Include numbers along axes only for bottom left plot of group (a 'group' is an analysis of identical markers).

☒ All plots are contour plots with outliers or pseudocolor plots.

☒ A numerical value for number of cells or percentage (with statistics) is provided.

Methodology

Sample preparation	To evaluate the cytotoxicity of T cells, organoids were dissociated into single cells and plated (1×105 per well) in a 24-well plate in the absence of Matrigel 24 hours before co-culture. Pretreated T cells (2×106) were added to each plate. After 6 hours, tumor cells were obtained. Using an Annexin V Apoptosis Detection Kit (Dojindo), the cells were stained according to the manufacturer's instructions, and then resuspended in phosphate buffered saline (PBS). Cell apoptosis analysis was conducted using a Beckman CytoFLEX FCM (Beckman Coulter) with the software CytExpert 2.0. The proportion of apoptosis was calculated using FlowJo V10 (BD). Each experiment contained 3 replicates.<br>MC38 cells were plated in a 24-well plate and left overnight in the presence of 200ng/ml mouse IFN-γ (Peprotech). T cells were added to the tumor cells at a 20:1 ratio at 37°C and co-cultured for 6 hours. Using an Annexin V Apoptosis Detection Kit (Dojindo), the MC38 cells were stained according to the manufacturer's instructions and resuspended in PBS for cell apoptosis analysis using a Beckman CytoFLEX FCM with the software CytExpert 2.0. The proportion of apoptosis was calculated using FlowJo V10. Each experiment contained 3 replicates.
Instrument	Beckman CytoFLEX FCM.
Software	Data was collected using CytExpert 2.0 (Beckman Coulter), and the proportion of apoptosis was calculated using FlowJo V10.
Cell population abundance	After co-culture, T cells were gently removed by PBS-washing, and T cells in the cell suspension were further removed using ficoll in order to obtain tumor cells for apoptosis assays. During flow cytometry, we draw a gate to exclude dead cells and particles in a FSC-A x SSC-A plot. After that, we draw a couple of gates that help to discriminate when two or more cells are stuck together as they pass through the flow cell in a SSC-A x SSC-H plot. Finally, single tumor cells were used for apoptosis assays.
Gating strategy	After cleaning up issues related to bubbles, clogs, and air, we draw a gate to exclude dead cells and particles in a FSC-A x SSC-

Gating strategy	A plot. After that, we draw a couple of gates that help to discriminate when two or more cells are stuck together as they pass through the flow cell in a SSC-A x SSC-H plot. To help gating the apoptotic cells, we prepare negative control cells, PI-only-stained cells, Annexin V-only-stained cells and PI-Annexin V-stained cells as pre-experiment in a PE-APC plot according to the manufacturer's instruction (Dojindo). Cells with positive Annexin V staining with or without positive PI staining were defined as apoptotic cells.

☒ Tick this box to confirm that a figure exemplifying the gating strategy is provided in the Supplementary Information.
